# Causal relationships between delirium and Parkinson’s disease: a bidirectional two-sample Mendelian randomization study

**DOI:** 10.1186/s40001-024-01696-9

**Published:** 2024-02-09

**Authors:** Xiaoliang Bai, Anna Zhang, Qingbo Zhou, Hongli Wang

**Affiliations:** 1 Department of Pain Management, The Second Hospital of Shandong University, Jinan, 250033 China; 2 Department of Geriatrics, South Branch of The Second Hospital of Shandong University, Jinan, 250033 China; 3 Department of Neurology, The Second Hospital of Shandong University, Jinan, 250033 China

**Keywords:** Parkinson’s disease, Delirium, Mendelian randomization, Causal relationships

## Abstract

**Background:**

Previous observational studies have suggested a notably elevated prevalence of delirium in individuals diagnosed with Parkinson’s disease (PD), thereby implying a potential increased susceptibility to delirium among PD patients. However, it is imperative to acknowledge that observational studies inherently possess limitations, rendering it arduous to establish a definitive causal or reverse causal association between delirium and PD.

**Methods:**

To explore the relationship between delirium and PD, a bidirectional two-sample Mendelian randomization (MR) was conducted using summary statistics obtained from genome-wide association studies. The main analysis was performed using the inverse-variance weighted (IVW) method, with further analyses conducted using MR Egger, weighted median, and weighted mode to ensure accuracy of findings. Additionally, Cochran’s Q statistics and MR Egger intercept were utilized to assess heterogeneity and horizontal pleiotropy, respectively.

**Results:**

According to the results obtained from the IVW model, no compelling evidence was found to support a potential causal association between delirium and PD (IVW: odds ratio [OR]: 0.996, 95% confidence interval CI 0.949–1.043, *P* = 0.845). Additionally, in the reverse direction, based on the results obtained from the IVW model, no significant evidence was found to support a causal association between PD and delirium (IVW: OR: 1.078, 95%CI  0.960–1.204, *P* = 0.225). A sensitivity analysis verified the reliability of the results.

**Conclusion:**

According to the MR findings, a bidirectional causal relationship between delirium and PD is not observed. It is crucial to conduct further research in clinical practice to investigate the association between delirium and the risk of PD.

**Supplementary Information:**

The online version contains supplementary material available at 10.1186/s40001-024-01696-9.

## Introduction

Delirium is an acute state of confusion characterized by inattention, disorganized and incoherent thinking, and aberrant perceptual function [1, 2]. Delirium is associated with an increased risk of falls, cognitive decline, morbidity and mortality [3, 4]. Moreover, delirium is an important non-motor function that has received increasing attention in Parkinson's disease (PD) and other forms of Parkinsonism [4]. Delirium has been commonly associated with parkinsonism, surpassing its occurrence in the general aging population [4, 5]. However, the existing research on delirium as a risk factor for PD remains limited, and there is a lack of studies investigating the prevalence of delirium specifically in PD patients [6]. Furthermore, no research has yet explored a causal relationship between delirium and PD. In addition, the limitations of observational studies [1], such as the inability to fully control for potential confounding variables, small sample sizes, and selection bias, pose challenges in establishing a definitive causal relationship between delirium and PD. Therefore, novel research approaches are essential to gain a comprehensive understanding of this causal association.

Recently, the utilization of large-scale genome-wide association study (GWAS) data and substantial sample sizes has introduced Mendelian Randomization (MR) as a powerful analytical method. MR employs genetic variants, typically single nucleotide polymorphisms (SNPs), as instrumental variables (IVs) to estimate the causal relationship between an exposure and a disease [7, 8]. It addresses issues of confounding and reverse causality more effectively, resembling the randomized controlled trial design due to the random assortment and combination of alleles during gamete formation [7], MR studies offer a higher level of evidence compared to observational studies [9]. In this study, we conducted a bidirectional two-sample MR analysis utilizing GWAS databases to systematically investigate the genetic causality between delirium and the risk of PD.

## Materials and methods

### Study design and MR assumptions

**Fig. 1:**
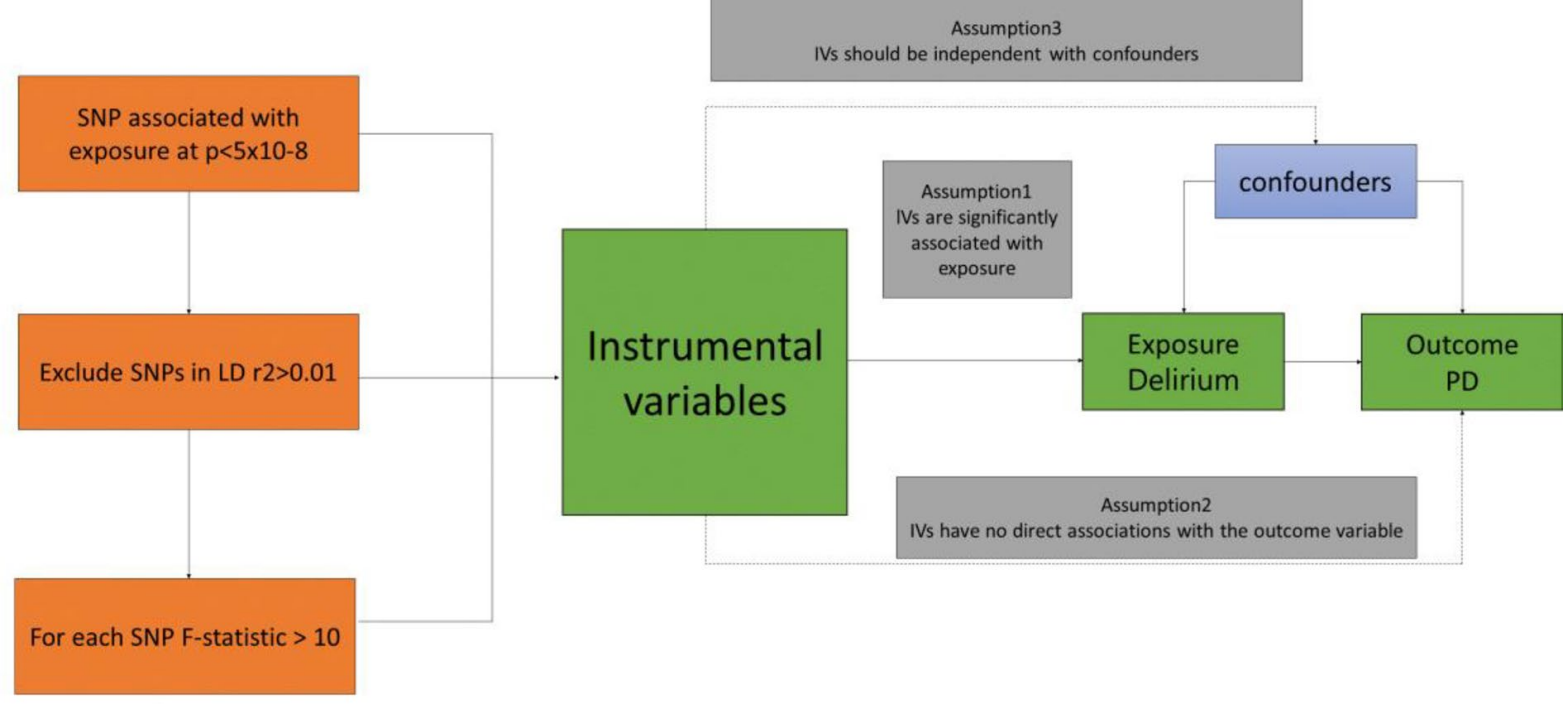
Overall design of Mendelian randomization analyses

To investigate bidirectional associations between delirium and PD through MR studies, we applied three fundamental assumptions to genetic variants [10]: (1) the assumption of association, which states that SNPs are closely linked to the exposure; (2) the assumption of independence, implying that SNPs are free from confounders along the exposure–outcome pathway; and (3) the assumption of exclusivity, suggesting that SNPs exclusively influence the outcome through exposure and not via other pathways. Figure 1 provides an overview of our study design.

### Data source

For our study, we sourced GWAS summary data on delirium from the FinnGen Consortium. The dataset consisted of 2612 delirium cases and 325,306 controls, all of Finnish ancestry. In total, we identified 21,168,109 SNPs in the analysis [1].

For the identification of genetic variants associated with PD prevalence, we analyzed data from a large-scale GWAS meta-analysis conducted by the International Parkinson’s Disease Genomics Consortium (IPDGC) for PD phenotypes, which comprised 33,674 cases and 449,056 controls [11].

### Selection criteria for IVs

In accordance with the core assumptions of MR studies, we included SNPs with correlations satisfying *P* < 5 × 10^–8^ as instrumental variables by screening the GWAS data. To mitigate the impact of linkage disequilibrium (LD) on analysis results, we enforced the condition of *r*^2^ < 0.001 and window size = 10,000 kb [12]. To ensure robust associations between instrumental and endogenous variables and to prevent weak instrumental variable bias, we calculated R^2^ [R^2^ = 2 × EAF × (1 − EAF) × b^2^], representing the proportion of variation explained by instrumental variable SNPs, and the F statistic [F = R^2^ × (N − 2)/(1 − R^2^)], used to evaluate the strength of instrumental variables, for each SNP separately [13, 14].

In addition to the previous information provided, we identified SNPs that were specifically associated with the outcome through exposure using the PhenoScanner (V2) database. This database is available at http://www.phenoscanner.medschl.cam.ac.uk/.

### Mendelian randomization study and sensitivity analysis

In this MR study, we primarily employed the inverse-variance weighted (IVW) method to explore the causal relationship between delirium and PD. To ensure the robustness of our statistical findings, we conducted sensitivity analyses using both the weighted median (WM) and Mendelian randomization-Egger regression (MR-Egger) based on Egger regression. The IVW method is considered the standard approach for MR pooled data [15], utilizing the Wald ratio method to estimate the causal effect for each included instrumental SNP [15]. The weighted median estimation method requires that at least 50% of the weights contributed by genetic variation are valid for statistical calculations [16]. MR-Egger regression identifies and corrects for multicollinearity, provided that the included instrumental variables satisfy the instrument strength independent of direct effect (INSIDE) assumption, which assumes independence between instrument-exposure and instrument-outcome associations [17]. Furthermore, weighted median [16] and maximum likelihood [18] methods were employed as complementary approaches to assess potential causality.

For sensitivity analyses, we calculated Cochran’s Q statistic using both IVW and MR-Egger regression. A *P*-value > 0.05 indicates no significant heterogeneity. Additionally, we employed the leave-one-out method, systematically excluding each included SNP one by one, and generated forest plots. A *P*-value > 0.05 after excluding a SNP suggests that the SNP does not significantly affect the results [15]. To assess pleiotropy, we used both the intercept term of MR-Egger regression and the Mendelian randomization pleiotropy residual sum and outlier (MR-PRESSO) test for the included SNPs. In MR-Egger regression, an intercept trending towards zero indicates the absence of horizontal pleiotropy. The MR-PRESSO test calculates the degree of influence of included instrumental variables and assesses the effect size between exposure and outcome after removing outliers, thereby allowing a pre- and post-correction comparison of results [19]. In this MR analysis, odds ratio (OR) served as the effect value, and a 95% confidence interval (CI) was applied. Statistical significance was considered at *P* < 0.05. The R 4.0.3 software, along with the two-sample-MR [20] and MR-PRESSO [19] packages, were used for data processing and visualization.

## Results

### Effect of delirium on Parkinson’s disease

Eight SNPs were identified as IVs in this study, following the exclusion of palindromic SNPs and SNPs associated with confounding factors. Notably, all of these selected SNPs yielded F-statistic scores exceeding 10, indicating a minimal risk of weak-instrument bias.

**Fig. 2:**
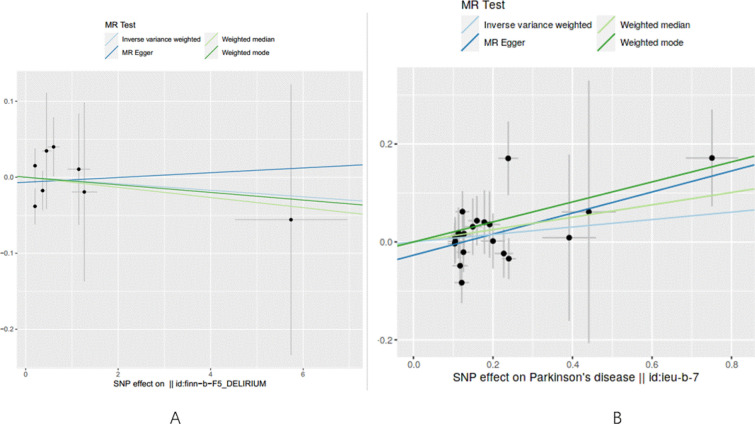
Scatter plot of genetic correlation between PD and delirium using four MR methods.** A** Evaluation the effect of delirium on PD.** B** Evaluation the effect of PD on delirium. *PD* Parkinson’s disease, *MR* Mendelian randomization

**Fig. 3:**
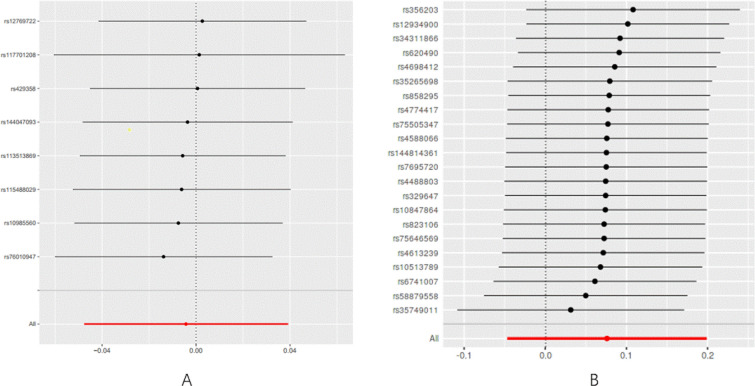
Leave-one-out analysis of the MR results between PD and delirium.** A** Delirium on PD.** B** AD on delirium. *PD* Parkinson’s disease, *MR* Mendelian randomization

According to the results obtained from the IVW model, no compelling evidence was found to support a potential causal association between delirium and PD (IVW: odds ratio [OR]: 0.996, 95% confidence interval CI 0.949–1.043, *P* = 0.845). Consistent findings were observed across other MR methods, including MR Egger (OR: 1.003, 95% CI 0.950–1.060, *P* = 0.916), weighted median (OR: 0.993, 95% CI 0.939–1.050, *P* = 0.816), and weighted mode (OR: 0.995, 95% CI 0.938–1.051, *P* = 0.864). A comprehensive overview of these results is presented in Table 1 and Fig. 2A. Furthermore, our analyses demonstrated no evidence of heterogeneity (Q = 4.82, *P* = 0.567) or horizontal pleiotropy (*P* = 0.694), as indicated in Table 2. Additionally, the results from the leave-one-out sensitivity analysis provided further support, illustrating that the causal effect was not driven by any single SNP (Fig. 3A).

**Table 1:** The result of the MR study and reverse MR study

Exposure	Outcome	Method	SNP (n)	β	se	*P*-value	OR (95CI%)
Delirium	PD	MR Egger	8	0.003	0.028	0.916	1.003 (0.950–1.060)
		Weighted median	8	− 0.006	0.028	0.816	0.993 (0.939–1.050)
		Inverse variance weighted	8	− 0.004	0.022	0.845	0.996 (0.949–1.043)
		Weighted mode	8	− 0.005	0.028	0.864	0.995 (0.938–1.051)
PD	Delirium	MR Egger	22	0.215	0.132	0.120	1.241 (0.795–1.684)
		Weighted median	22	0.126	0.088	0.153	1.135 (0.891–1.391)
		Inverse variance weighted	22	0.076	0.062	0.225	1.078 (0.960–1.204)
		Weighted mode	22	0.204	0.141	0.162	1.226 (0.915–1.597)

*OR*, odds ratio, *CI* confidence interval, *MR* Mendelian randomization, *SNPs* single nucleotide polymorphisms, *NA* not available

^*^The statistically significant difference with a *P*-value less than 0.05 (*P* < 0.05)

**Table 2:** Heterogeneity and horizontal pleiotropy analysis between PD and delirium

Exposure	Outcome	Heterogeneity				Horizontal pleiotropy	
		MR Egger		Inverse variance weighted		MR Egger intercept	
		Q	*P* value	Q	*P* value	SE	*P* value
Delirium	PD	4.82	0.567	4.99	0.661	0.016	0.694
PD	Delirium	16.03	0.714	17.45	0.683	0.023	0.247

### Effect of Parkinson’s disease on delirium

Twenty-two SNPs were identified as IVs after excluding palindromic SNPs and those associated with confounding factors. Of particular note, the selected SNPs displayed F-statistic scores greater than 10, demonstrating a minimal risk of weak-instrument bias.

Based on the results obtained from the instrumental variable-weighted (IVW) model, no significant evidence was found to support a causal association between PD and delirium (IVW: odds ratio [OR]: 1.078, 95% confidence interval CI 0.960–1.204, *P* = 0.225). Consistent findings were observed across other MR methods, including MR Egger (OR: 1.241, 95% CI 0.795–1.684, *P* = 0.120), weighted median (OR: 1.135, 95% CI 0.891–1.391, *P* = 0.153), and weighted mode (OR: 1.226, 95% CI 0.915–1.597, *P* = 0.162). Table 1 and Fig. 2B provide a comprehensive overview of these results. Our analyses also revealed no evidence of heterogeneity (Q = 16.03, *P* = 0.714) or horizontal pleiotropy (*P* = 0.247), indicating that the associations were not confounded by pleiotropic effects, as noted in Table 2. Additionally, the leave-one-out sensitivity analysis demonstrated that the causal effect was not driven by any individual SNP, further supporting the validity of the results (Fig. 3B).

## Discussion

The causal relationship between delirium and PD lacks a comprehensive understanding, necessitating further investigation. This study aims to address this research gap by examining the causal association between delirium and PD using a bidirectional two-sample MR analysis. Notably, to the best of our knowledge, this study represents the first attempt to explore the genetic risk aspect of this relationship. Moreover, our findings suggest no significant genetic susceptibility correlation between delirium and PD.

In this investigation, we employed a range of MR analysis techniques to uncover compelling evidence that challenges the notion of delirium as an autonomous risk factor for PD. The consistency of our findings was bolstered by the absence of any pleiotropy or heterogeneity in the sensitivity analyses, rendering them highly credible. However, while we cannot wholly dismiss the possibility that delirium may impact the progression of PD via comparable pathogenic mechanisms, such as neurotransmission abnormalities and neuroinflammation, it is vital to acknowledge that further research is warranted. Future studies with larger sample sizes are needed to verify the influence of delirium on the course of PD and to elucidate the true underlying drivers that contribute to its acceleration.

Parkinson’s disease is recognized as a risk factor for developing delirium, but the prevalence has been found to vary widely, with a range of prevalence of 0.3%–60 in studies in different settings [21–23]. The overlapping symptoms between PD and delirium create diagnostic difficulties [5]. Delirium is commonly classified based on motor subtypes, namely hypoactive, hyperactive, or mixed [24, 25]. Hypoactive delirium is characterized by a reduction in psychomotor activity [26]. Cullinan et al [27] found that delirium in patients with PD is common but often missed, especially in the hypoactive delirium subtype. Hence, it is crucial for clinicians and caregivers to meticulously identify susceptible risk factors for delirium in patients with PD and implement timely preventive measures. By doing so, there is a greater likelihood of early recognition and effective prevention of delirium.

Extensive research has recently focused on the causal relationship between delirium and PD [28, 29]. Some potential mechanisms may partly explain such association. The inflammatory response has been widely recognized as a significant contributor to acute brain dysfunction or delirium. Moreover, critical illness accompanied by acute inflammatory injury has been identified as a risk factor for PD [30–32]. A meta-analysis of 152 observational studies has revealed elevated levels of PD-associated biomarkers, such as IL-6 and C-reactive protein [33]. The presence of shared biomarkers between delirium and PD suggests potential overlapping pathological mechanisms during disease progression [27]. Furthermore, these delirium-related biomarkers may also contribute to the detrimental course of PD, rather than solely being indicative of delirium itself. Moreover, vitamin D deficiency, may lead to delirium and PD. Previous MR analysis and cohort studies have found an association between low vitamin D concentrations and the onset of delirium [34–36]. Previous studies have extensively documented the markedly high incidence of vitamin D deficiency among individuals suffering from PD and the notable predictive capacity of such deficiency for both the onset and progression of this condition [37–39]. Hence, we postulate that there exists a plausible association between vitamin D deficiency and an augmented susceptibility to delirium among PD patients.

However, it is important to note that our study specifically focused on evaluating the causal relationship between delirium and PD among patients with or without PD. It’s important to note that the scope of our study does not encompass the course and progression of PD. While our findings did not indicate an increased risk of developing PD in relation to delirium, it is crucial to emphasize that this conclusion does not contradict the notion that delirium can potentially accelerate the risk of developing PD. Subsequent investigations should incorporate more extensive sample sizes to validate the influence of delirium on the progression of PD and to delve into the genuine underlying factors that facilitate the acceleration of PD progression (Additional file 1).

This study presents several notable research advantages. Firstly, the implementation of the MR model effectively addresses confounding variables and reverse causation, providing more robust causal effect estimates compared to standard observational studies [40]. Additionally, the use of a large-sample GWAS dataset greatly enhances the statistical power compared to smaller sample sizes relying on individual data [15]. Secondly, the MR approach allows for simultaneous control of instrumental variable errors associated with both the exposure and outcome, while also accounting for bias introduced by linkage disequilibrium among instrumental variables [15]. Lastly, bidirectional MR studies have the distinct advantage of circumventing the effects of reverse causation and minimizing residual confounding.

Nevertheless, there are several limitations in our study. Firstly, since all data were sourced from individuals of European descent, the results may not be generalizable to populations of different ethnic backgrounds. Secondly, due to the unavailability of gender- or age-stratified data in the GWAS datasets used, we were unable to assess whether the associations between delirium and PD differ across gender or age groups. Further research should explore these potential variations when stratified GWAS pooled data become accessible. Third, despite the comprehensive sensitivity analyses conducted to test MR study hypotheses, complete elimination of the possibility of horizontal pleiotropy among instrumental variables remains challenging [41]. Moreover, differences in gene annotation analysis platforms across GWAS cohort studies may have contributed to the heterogeneity of this study [42]. Notably, our study did not incorporate an evaluation of delirium or its varying levels of severity. Consequently, we were unable to examine the association between PD and specific subtypes or the severity of delirium. This omission regarding delirium subtype or severity highlights the need for future research endeavors to address these critical factors. By incorporating such variables into the analysis, a more comprehensive understanding of the interplay between PD and delirium can be attained.

## Conclusions

Our bidirectional two-sample MR analysis showed no bidirectional causal relationship between delirium and PD. Nonetheless, future studies are needed to explore the potential mechanisms of the effect of PD on delirium, as well as to utilize larger sample sizes to confirm the effect of delirium on PD.

### Supplementary Information

**Additional file 1: Figure S1.** Forest plot of the MR results between PD and delirium. **A** Delirium on PD. **B** PD on delirium, *MR* Mendelian randomization. **Figure S2.** Funnel plots of the association between PD and delirium. **A** Delirium on PD. **B** AD on delirium, *MR* Mendelian randomization.

## Data Availability

The GWAS summary data for PD (ieu-b-7) and Delirium (finngen-R8-F5_DELIRIUM) are publicly available, and can be downloaded from GWAS (https://gwas.mrcieu.ac.uk/) and FinnGen website (https://www.finngen.fi/en).

## Abbreviations

PD: Parkinson’s disease
MR: Mendelian randomization
IVW: Inverse-variance weighted
SNPs: Single nucleotide polymorphisms
GWAS: Genome-wide association study
LD: Linkage disequilibrium
OR: Odds ratio
