# Supplementary material for: Causal relationships between delirium and Parkinson’s disease: a bidirectional two-sample Mendelian randomization study
Source: Eur J Med Res. 2024 Feb 9;29:111. doi: 10.1186/s40001-024-01696-9 (PMC10854158; doi:10.1186/s40001-024-01696-9)
Supplement: Supplementary file 1 — Additional file 1: Figure S1. Forest plot of the MR results between PD and delirium. A Delirium on PD. B PD on delirium, MR Mendelian randomization. Figure S2. Funnel plots of the association between PD and delirium. A Delirium on PD. B AD on delirium, MR Mendelian randomization. [file 40001_2024_1696_MOESM1_ESM.docx]

**Supplementary Material**

**
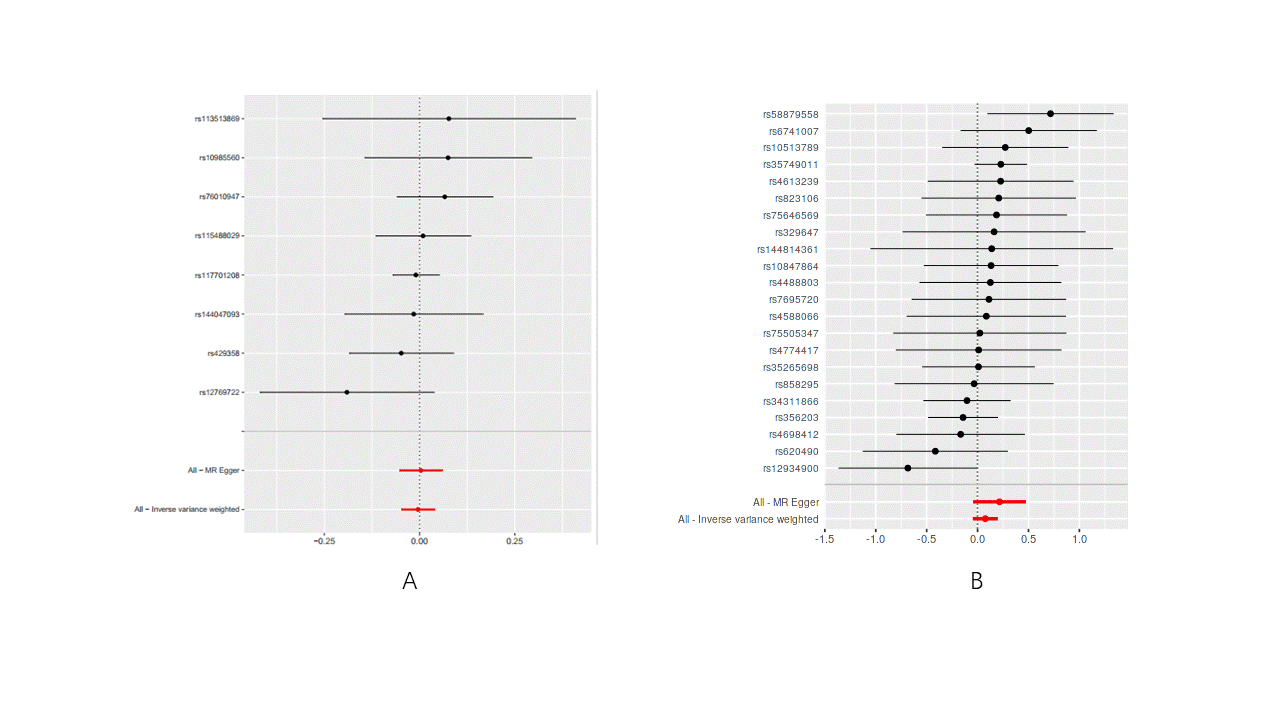
**

**Figure S1：**Forest plot of the MR results between PD and delirium. A: Delirium on PD. B: PD on delirium, MR: Mendelian randomization


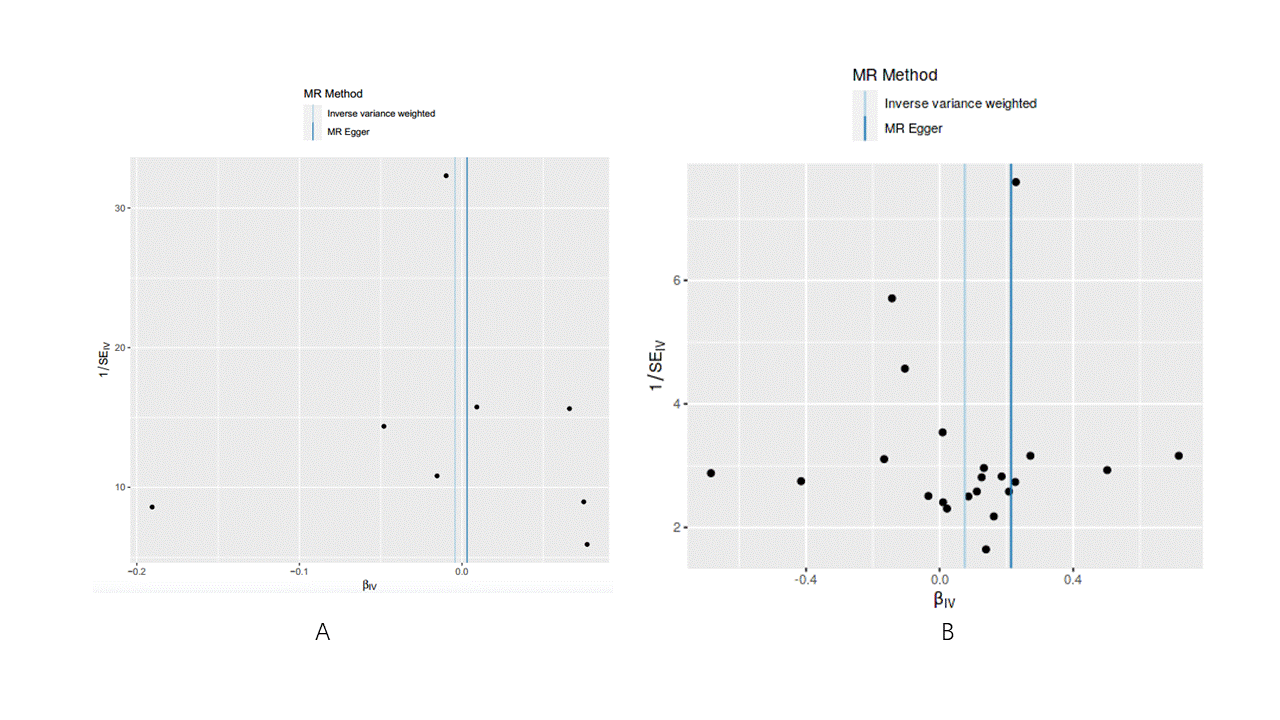


Figure S2： Funnel plots of the association between PD and delirium. A: Delirium on PD. B: AD on delirium, MR: Mendelian randomization
